# Topological classification of driven-dissipative nonlinear systems

**DOI:** 10.1126/sciadv.adt9311

**Published:** 2025-08-13

**Authors:** Greta Villa, Javier del Pino, Vincent Dumont, Gianluca Rastelli, Mateusz Michałek, Alexander Eichler, Oded Zilberberg

**Affiliations:** ^1^ Department of Physics, University of Konstanz, 78464 Konstanz, Germany.; ^2^ Laboratory for Solid State Physics, ETH Zürich, 8093 Zürich, Switzerland.; ^3^ Quantum Center, ETH Zürich, 8093 Zürich, Switzerland.; ^4^ Pitaevskii BEC Center, CNR-INO and Dipartimento di Fisica, Università di Trento, I-38123 Trento, Italy.; ^5^ INFN-TIFPA, Trento Institute for Fundamental Physics and Applications, Via Sommarive 14, I-38123 Trento, Italy.; ^6^ Department of Mathematics and Statistics, University of Konstanz, 78457 Konstanz, Germany.

## Abstract

In topology, averaging over local geometrical details reveals robust global features. These are crucial in physics for understanding quantized bulk transport and exotic boundary effects of linear wave propagation in (meta-)materials. Beyond linear Hamiltonian systems, topological physics strives to characterize open (non-Hermitian) and interacting systems. Here, we establish a framework for the topological classification of driven-dissipative nonlinear systems by defining a graph index for their Floquet semiclassical equations of motion. Our index builds upon the topology of vector flows and encodes the particle-hole nature of excitations around all out-of-equilibrium stationary states. Thus, we uncover the topology of nonlinear resonator’s dynamics under external and parametric forcing. Our framework sheds light on the topology of driven-dissipative phases, including under- to overdamped responses and symmetry-broken phases linked to population inversion. We therefore expose the pervasive link between topology and nonlinear dynamics, with broad implications for interacting topological insulators, topological solitons, neuromorphic networks, and bosonic codes.

## Teaser

A graph index reveals topological structures in driven-dissipative nonlinear systems.

## INTRODUCTION

**Fig. 1.**
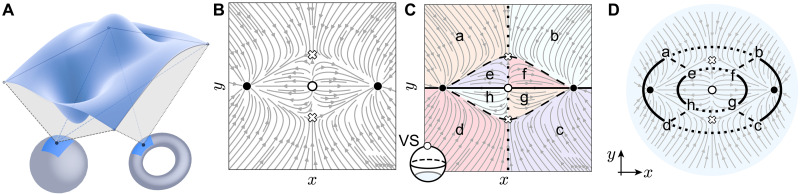
Vector flow topology. (**A**) Topographic landscape corresponding to a 2D surface with a height function. In topology, we average over the local geometric curvature to determine the global shape of a manifold, e.g., a sphere or a torus. (**B**) The topography can be mapped to a gradient vector flow (compare to Eq. 1) with peaks, valleys, and passes, corresponding to sources (white dot), sinks (black dot), and saddles (white cross), respectively. (**C**) A MSC splits the manifold into regions (cells) with different behavior of flow lines. The regions are separated by lines that connect sources to saddles (dotted), saddles to sinks (dashed), and sources to sinks (solid). Lowercase letters label each region. (inset) The MSC skeleton or graph is defined on a compact manifold by embedding the flow around the south pole of a sphere and adding a virtual source (VS) at the top, from which the flow originates (*3*, *4*). (**D**) The MSC graph is associated with a colored-graph topological invariant (*3*, *4*): Nodes mark MSC cells (lowercase letters) and edges connect neighboring cells. The edge dashing matches the dashing of the cell boundaries of the MSC. The resulting three-dashed graph [equivalent to the three-colored graph in (*4*)] uniquely encircles regions corresponding to each critical point and classifies their spatial arrangement.

When we travel on Earth, we experience changes in its surface elevation f(x,y) depending on our local latitude and longitude coordinates *x* and *y*, respectively. These changes reflect local curvature variations due to mountains, valleys, and passes; see Fig. 1A. To determine Earth’s global shape, we integrate over the local curvature and obtain a topological index (Euler characteristic) that classifies our planet as a sphere and not a torus (*1*, *2*). The global shape and its topological invariant are robust to local landscape distortions. Crucially, “topology of topography” goes beyond the classification of global shapes and can discern between nonlocal arrangements of landscape features, e.g., between networks of watersheds (*3*–*5*). The latter discerns which basins are filled with rainfall, as water flows downhill along a gradient flow; see Fig. 1B. By relation of the topography to a two-dimensional (2D) vector flow, we can use vector field topology (*6*, *7*) to classify the watersheds’ arrangement. The classification relies on a Morse-Smale complex (MSC) (Fig. 1C) or a related Morse-Smale graph index (Fig. 1D) that encapsulates the numbers, types, and arrangement of mountains, valleys, ridges, and passes. Notably, the flow pattern and the topological index are robust against small perturbations in the topographic structure due to structural stability (*3*). Vector field topology has wide-ranging applications in various fields, including solar flares, data compression, computer vision, shape analysis, weather forecasting, and robotics (*8*, *9*). Related approaches are also used in classifying different types of chaos, where the focus shifts to the internal flow structure of strange attractors (*10*, *11*).

Topology also plays a key role in wave propagation through media (*12*). The transport pertains to bulk energy bands that are characterized by topological indices with associated robust quantized responses and corresponding exotic boundary modes. The topological classification of electron waves has revealed a plethora of previously unknown materials (*12*, *13*) and prompted their demonstration using a variety of photonic (*14*), mechanical (*15*), electronic (*12*), and atomic (*16*) metamaterial emulators. As the latter commonly involve driven-dissipative resonators with linear bosonic excitations, recent research deals with the topological classification of non-Hermitian dynamics and the associated winding of complex energy bands around exceptional points (*17*, *18*). In nonlinear systems, the variety of competing effects and many-body phase transitions makes topological classification much more involved (*19*, *20*). Commonly, only the linear excitations on top of specific nonlinear many-body states are studied using the approaches detailed above (*21*–*27*). The band topology of these linear excitations provides local information and is blind to the richer variety of states the system may host. Therefore, a comprehensive topological classification of the rich combination of driving, dissipation, and many-body interactions remains elusive.

Here, we establish a general framework for the topological classification of driven-dissipative nonlinear systems. We analyze the Floquet (rotating) semiclassical equations of motion and use vector field topology to define a unique graph index that discerns between different arrangements of nonlinear nonequilibrium stationary states (NESS). Specifically, our index describes the structural arrangement of attractors (sinks) and repellors (sources) in the rotating phase space and incorporates the particle-hole characteristics of excitations around each NESS. Accordingly, our index reflects global characteristics spanning the full range of NESSs rather than local traits near a specific state. In a microelectromechanical nonlinear resonator experiment, we measure the different graph indexes arising when subject to both single- and two-phonon drives. Our framework captures the topological origin of locking to different drives, over-to-underdamped dissipative transitions, and population inversions in the system. We thus pave the path for a comprehensive unveiling of topological effects in nonlinear media (*28*–*31*) and in driven-dissipative collective phenomena (*32*–*36*).

We first recapitulate the topological analysis of vector fields (*8*, *9*). Consider, for example, a 2D real vector flow

$$\dot{r}=-\nabla f\left(r,\mu \right)$$ (1)

generated by the gradient of a potential function *f*, polynomial in coordinates r=(x,y) , with μ as additional parameters. Structural stability in the 2D flow (*1*) requires that (*3*): (i) each trajectory ends in a nondegenerate singular point or a limit cycle, (ii) the flow contains finitely many singular points, i.e., points where $\dot{r}=0$ (the critical points of *f*), and (iii) no trajectories exist between saddles. If these conditions hold, trajectories in the flow can be smoothly transformed into each other via perturbations of the parameters μ , while preserving the overall flow pattern, i.e., maintaining topological equivalence between the flows. Conversely, a modification that leads to a different flow pattern requires breaking of structural stability as a function of μ , marking a topological phase transition. The flow pattern is captured by an MSC (*37*), comprising a graph with critical points as nodes, separatrices as edges, and resulting encapsulated cells; see Fig. 1C.

Structurally stable systems in 2D admit a unique, topological invariant tied to its MSC (*4*). Such an invariant consists of a graph index where the edges are three-colored, provided that the manifold with coordinates r is compact, and no limit cycles are present; see Fig. 1D. This graph index is unique for each topological phase (*4*). In our case, we use dashing instead of colors to distinguish the edges. Isomorphic graphs have matching connectivity and dashing, indicating topologically equivalent flows. Such vector flows therefore belong to the same, structurally stable, “topological phase.” A graph-changing topological phase transition occurs when (A) the number *N* of NESS changes via bifurcations; (B) the counts of saddles $\left(N_{s}\right)$ , minima $\left(N_{m}\right)$ , and maxima $\left(N_{M}\right)$ change while preserving $N\left(N\equiv N_{s}+N_{m}+N_{M}\right)$ ; or (C) separatrices form or annihilate. Condition (A) is insensitive to the global flow topology in phase space as it relates to the linear stability analysis near a critical point. In contrast, condition (C) requires a global phase-space analysis, enabling a classification of the overall dynamics. Since separatrices form and vanish when critical points emerge or disappear (but not vice-versa), condition (A) can be viewed as a special case of (C). Note that parameter changes in μ that break the structural stability conditions, e.g., by creating a saddle connection, make the MSC and the graph invariant mathematically ill-defined.

## RESULTS

### Topology of nonequilibrium vector flows

**Fig. 2.**
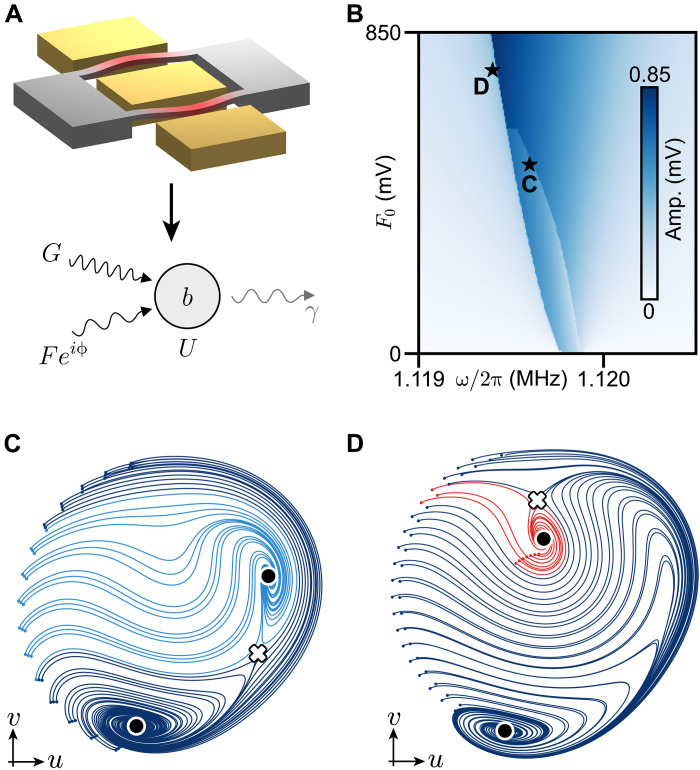
Driven-dissipative NESS of a nonlinear resonator. (**A**) Top, sketch of the microelectromechanical resonator used in this work (*38*, *41*). The vibrations are driven, and the rotating quadratures *u* and *v* at ω are detected via two electrodes; see Materials and Methods. Bottom, model illustration (compare to Eq. 2) of a driven-dissipative bosonic mode *b* subject to one-phonon (*F*) and two-phonon (*G*) drives. The mode has nonlinearity *U* and experiences phonon loss with rate γ . (**B**) Stationary amplitude for a red-to-blue frequency sweep of both drives as a function of $F\propto F_{0}$ for a fixed $G\propto G_{0}\approx 3.2V$ and phase ϕ≈0.47π ; see Materials and Methods. (**C**) Rotating-frame ringdown reconstruction of the vector flow (*41*) at $F_{0}\approx 0.5V$ and ω/(2π)≈1.1196MHz . Blue line shades distinguish between clockwise spiraling ringdowns into two different stable NESS. (**D**) The same as (C) at $F_{0}\approx 0.75V$ and ω/(2π)≈1.1194MHz . Blue (red) lines indicate clockwise (counterclockwise) spirals into different stable NESSs.

We aim to apply this topological classification framework to driven-dissipative nonlinear systems, relevant for a variety of photonics, mechanics, electric, and cold atoms realizations (*28*–*36*). Here, we present an experiment using a microelectromechanical resonator (MEMS) (*38*, *39*) under single- and two-phonon drives at frequencies ω and 2ω and with strengths *F* and *G*, respectively (Fig. 2A). This device was chosen for its simplicity, needing no lasers, cryogenic cooling, or high voltages, while representing a common model in many nonequilibrium quantum-engineered platforms (*31*). We apply the driving via voltage tones with amplitudes $F_{0}\propto F$ and $G_{0}\propto G$ ; see Materials and Methods and (*40*, *41*). Using a lock-in measurement, we retrieve the quadrature amplitudes q=(u,v) of the resonator relative to a local oscillator at frequency ω , with x(t)=ucos(ωt)−vsin(ωt) . This establishes a reference clock that fixes the axes (gauge) for **q** in the rotating phase space (Materials and Methods and Supplementary Text) (*42*). For a fixed value of *F* and *G*, we measure the stationary amplitude of the system’s response for different (increasing) values of ω . This procedure results in a single horizontal line in Fig. 2B. Repeating this procedure for different values of *F* yields the entire diagram. Sharp features correspond to first-order phase transitions between different NESS (*30*, *43*–*45*). Crucially, a parameter sweep generally samples a subset of available stationary phases but does not capture the complete phase diagram of rotating vector flows in the system. For any particular set of parameters, however, we can reconstruct the slow time evolution of *u* and *v* using several ringdown measurements with variable initial conditions (*41*); see dots and experimental trajectories in Fig. 2 (C and D). For such ringdown measurements, we first initialize the system at a given point in phase space (see dots) by applying an additional large external force. Then, we switch off this force, allowing the system to follow a trajectory toward one of the solutions dictated by the one- and two-phonon drives. These trajectories are analogous to topographic vector flows (Fig. 1C), but in a rotating-frame phase space.

In the following, we will construct a graph invariant to classify the measured vector flows and obtain a topological phase diagram for the system. We note, however, that the measured vector flows differ from gradient flows introduced in Fig. 1. Specifically, the flows observed in Fig. 2 (i) have only saddles as unstable fixed points, (ii) lack sources, and (iii) feature sinks with clockwise/counterclockwise spirals, corresponding to faster/slower dynamics than the reference oscillator (*41*). To assess the topological significance of the observed differences, we write the effective Hamiltonian of the system in a rotating frame at frequency ω using a Floquet expansion technique based on counting driving phonons (Supplementary Text) (*46*)

$$\begin{array}{c} H=\left(-\Delta +U\right)b^{†}b+\frac{U}{2}b^{†2}b^{2}-\frac{G}{2}\left(b^{2}e^{i\psi }+b^{†2}e^{-i\psi }\right)-F\left({\mathrm{be}}^{i\phi }+b^{†}e^{-i\phi }\right) \end{array}$$ (2)

Here, the operator b annihilates phonons in the resonator, defined as $b=\sqrt{\omega /2}\left(X+\mathrm{iY}\right)$ and *ħ* = 1. The quadrature operators satisfy 〈X〉=u and 〈Y〉=−v , with the commutation relation [X,Y]=2i/ω ; see Supplementary Text. Furthermore, *U* parametrizes phonon-phonon interactions, and $\Delta =\left(\omega^{2}-\omega_{0}^{2}\right)/\left(2\omega \right)$ is the detuning between the natural frequency of the resonator $\omega_{0}/\left(2\pi \right)\approx 1.1198\text{MHz}$ and drive frequency ω , compare to Fig. 2A. We use a fixed two-phonon driving phase ψ = 0, making ϕ the phase difference between the single- and two-phonon drives. Parameters *U*, *G*, and F map to the MEMS Duffing nonlinearity $k_{3}\left(k_{3}\approx -9.9\times 10^{16}V^{-2}s^{-2}\right)$ and the voltage amplitudes $G_{0}$ and $F_{0}$ of the applied tones, respectively; see Materials and Methods. The experiment operates in the semiclassical limit due to weak interactions (*U*) and high phonon population $\left(\langle b^{†}b\rangle \gg 1\right)$ , allowing us to use the mean-field approximation 〈AB〉≈〈A〉〈B〉 . In addition, we add the phonon dissipation rate γ/(2π)≈112Hz such that the rotating quadratures follow the flow

$$\dot{q}=-J\nabla \tilde{H}\left(q\right)-\nabla R\left(q\right),\text{where}\;J=\left[\begin{array}{cc} 0 & -1\\ 1 & 0 \end{array}\right]$$ (3)

and the semiclassical Hamiltonian potential $\tilde{H}\left(q\right)$ is obtained by replacing b→〈b〉 in Eq. 2. In the rotating frame, dissipation enters as a Rayleigh potential of the form $R\left(q\right)=\gamma {|q|}^{2}/4$.

**Fig. 3.**
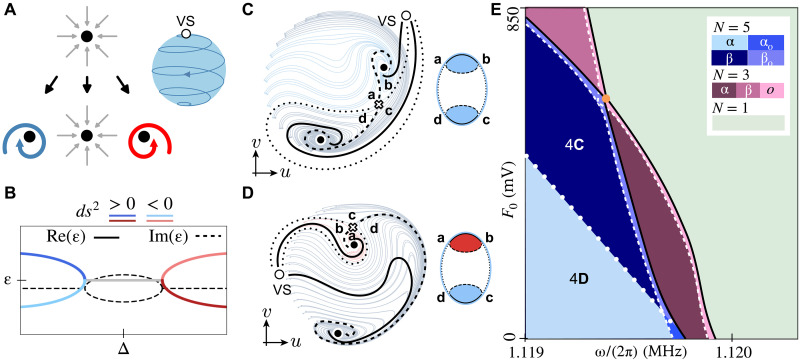
Topological invariant for driven-dissipative nonlinear systems. (**A**) Sinks are mapped to attractive foci with positive (blue), negative (red), or no chirality (no color). We embed the flow on a sphere using an inverse stereographic projection while keeping all critical points at the southern hemisphere; see Materials and Methods. The infinity is mapped to a virtual source (VS) at the top. As the nonlinearity dominates the flow at high amplitudes, it acts as a clockwise source (for U<0 ) at the north pole. (**B**) Exemplary complex Bogoliubov excitation spectrum ε around a NESS (compare to Eq. 4), where the real (imaginary) part are frequencies (inverse lifetime). Particle (hole-)-like excitations, discerned by a positive (negative) symplectic norm ${\mathrm{ds}}^{2}$ , correspond to clockwise (counterclockwise) chiralities (*41*). Exceptional points mark transitions between underdamped and overdamped excitations. (**C** and **D**) The reconstruction of the topological graph index for the measured flows in Fig. 2 (C and D, respectively). Cell colors mark local chirality, and the halo around the graphs highlights background chirality. The separatrix lines in (C) and (D) are simplified for illustration purposes as they tend to whirl rapidly near the attractors and become visually dense. (**E**) Full topological phase diagram of the system (compare to Eq. 2 and Fig. 2B). Hue indicates solutions’ count; shades denote chirality or separatrix connectivity (see legend). Solid, dashed, and dotted lines mark transitions in solution number, exceptional points, and separatrix connectivity changes, respectively. A multicritical point, where regions with N=1,3 and 5 meet is marked in orange. Regions related to Fig. 4 (C and D) are marked. For clarity, the width of the overdamped regions is exaggerated. Other parameters match those in Fig. 2.

In the symplectic limit (γ=0) , the flow (*3*) exhibits closed orbits orthogonal to $\tilde{H}$’s gradient flow. In the dissipative Rayleigh $\left(\tilde{H}=0\right)$ limit, the flow has a single sink at the origin. While standard Morse-Smale topological classification applies to the gradients flows of $\tilde{H}$ or of *R* independently, the combination in Eq. 3 cannot be mapped to a gradient-type flow. Consequently, sinks and sources in the flow are not straightforwardly linked to the critical points of the generating potentials. Sinks in Fig. 2 (C and D) form both at the maxima and minima of $\tilde{H}$ for small γ and can exhibit different flow chiralities; see Fig. 3A.

The local chirality of the flow around each NESS in Fig. 2 is linked to the particle-hole character of excitations (*32*, *41*); see Fig. 3B. Specifically, we analyze the bosonic excitations $\delta b=b-b_{0}$ around each NESS $b_{0}$ using the Bogoliubov–de Gennes Hamiltonian. It is defined as $H_{\text{BdG}}=JH$ , where H is the Hamiltonian matrix derived from the fluctuation Hamiltonian

$$\begin{array}{c} H_{\text{fl}}=\left[-\Delta +U\left(1+{|b_{0}|}^{2}\right)\right]\delta b^{†}\delta b+\frac{1}{2}\left[\left({\mathrm{Ub}}_{0}^{*2}-{\mathrm{Ge}}^{i\psi }\right)\delta b^{2}+H.c.\right] \end{array}$$ (4)

expressed in the basis $\left(\delta b,\delta b^{†}\right)$ . The dynamical matrix of the system then follows from $D=H_{\text{BdG}}-\left(\gamma /2\right)1$ to account for dissipation. The Bogoliubov excitations, found from the eigenstates of D , are ascribed a vector norm weighed with the metric *J* in Eq. 3. This so-called symplectic norm, ${\mathrm{ds}}^{2}$ , takes values $\text{sign}\left({\mathrm{ds}}^{2}\right)\in \left\{1,\emptyset ,-1\right\}$ that encode particle, overdamped, and hole excitations, respectively. Note that particle and hole excitations correspond to clockwise and counterclockwise chiralities (*41*, *47*). These chiralities are well-defined, as the drive frequency sets a fixed clock (gauge) that serves as the chirality reference. Overdamped excitations appear after an exceptional point, where the two lifetimes typically corresponding to squeezed and antisqueezed resonator quadratures, respectively. Alas, the MSC-based invariant in Fig. 1D overlooks this aspect, as the chirality of spirals can be flipped by a mirror reflection without altering the graph index. The excitations’ nature, however, is fixed by their relation to the reference oscillator (a gauge fixing). The latter restricts the allowed deformations of μ . Analogous to symmetry-protected topological phases (*48*), we can therefore topologically classify structurally stable vector flows under deformations that respect the chirality around all critical points. Mathematically, this implies that the allowed deformations must commute with transformations that change the symplectic norm around any NESS.

We encode the added symmetry constraint given by the symplectic norm into the graph invariant (compare to Fig. 1D) by the following construction: (i) We distinguish between sinks using colors based on $\text{sign}\left({\mathrm{ds}}^{2}\right)$ (compare to Fig. 3A). (ii) To handle flow lines extending to (from) infinity in the q plane, we embed the flow on a sphere, mapping these lines to a “virtual source” at the north pole; see Fig. 3A. Here, the dissipation potential *R* creates streams from the north to the south pole with a chirality dictated by the nonlinearity *U*, which we also decorate by colors and markers. (iii) The MSC complex, its linedashing styles, and the ensuing graph index are built using the same rules as in Fig. 1D, where we recall that we only have sinks and saddles in the presence of a single virtual source. (iv) We introduce colored faces to the graph index, indicating each of the NESS’s chirality $\text{sign}\left({\mathrm{ds}}^{2}\right)$ . The colored line or “halo” surrounding the planar graph encodes the chirality of the background flow, i.e., the asymptotic flow far from the NESS, which is determined by the system’s dominant nonlinearity (in our case, U<0 ). Two topological phases are equivalent if their graphs are isomorphic, preserving face colors, nodes, and edge dashing. We thus find that the measured flows in Fig. 2 (C and D) are topologically distinct; see Fig. 3 (C and D). Crucially, this distinction is commonly overlooked when counting only the total number *N* of stationary states.

Equipped with our graph index, we can now predict our resonator’s complete topological phase diagram; see Fig. 3E. It is generally split into three regions according to the number of critical points: *N* = 1 (1 stable solution), *N* = 3 (2 stable, 1 unstable), and *N* = 5 (3 stable, 2 unstable). We refer to phases with N=1 solution as monostable and those with N>1 solutions as multistable. Different topological phases split the diagram further into subparts, which we enumerate using Greek-letter subscripts, e.g., $N_{\alpha }$ . Topological phase transitions occur at all marked lines, corresponding to three types of possible effects: (a) Solid lines indicate transitions where the variety of NESS $\left(N_{s},N_{m},N_{M}\right)$ changes; (b) dashed lines mark under-to-overdamped transitions around a NESS; and (c) dotted lines mark graph connectivity changes that do not involve any of the changes (a) or (b). Notice that the dashing style of the transition lines has no correspondence to the dashing of the graph’s edges. Already by the number of solutions, we identify that regions *N* = 1 and *N* = 5 correspond to a standard driven harmonic oscillator phase and to a coexistence region between low- and high-amplitude phases in response to both the drives *F* and *G* (*30*, *31*, *44*, *49*). For *N* = 3, our classification draws a distinction between $3_{\alpha }$ and $3_{\beta }$ regions, signaling the graph-index variety exposed in Fig. 3 (C and D). In other words, our flow topology distinguishes between the Kerr parametric oscillator ( $3_{\alpha }$ ) and a Kerr driven oscillator ( $3_{\beta }$ ). Physically, in $3_{\alpha }$ , the resonator locks onto the parametric drive *G*, with flows showing two sinks of equal chirality split by a saddle. The flow is only perturbatively ${\mathbb{Z}}_{2}$ symmetry broken (*30*) by *F*. In $3_{\beta }$ , a standard Duffing bistable regime appears in response to *F*, with *G* acting as a perturbation (see Supplementary Text and fig. S1). Here, the resonator locks onto the single-phonon drive, with flows showing two sinks with opposite chiralities and a saddle. To preserve the topological distinction, all four phase boundaries converge at a multicritical point, located at the intersection of the solid lines, where a saddle and a sink annihilate and then reemerge as a new saddle and sink pair, with the sink’s chirality reversed. Since particle-like and hole-like attractors correspond to distinct excitation modes, this topological transition leaves clear signatures in the system’s linear response correlators (*32*). Note that our system can have a maximum of five fixed points (*50*, *51*).

Our graph invariant captures changes in the non-Hermitian excitations around each NESS, compare to flipped chirality between $3_{\alpha }$ and $3_{\beta }$ . The dashed boundaries in Fig. 3E capture transitions driven by the interplay between damping (loss) and squeezing (gain). Specifically, the interplay can modify the excitation around any NESS from being underdamped ( $\tilde{H}$ flow dominates) to overdamped (*R* flow dominates). Strong damping causes spirals to collapse into attracting nodes and lose chirality, marking dissipation-induced phase transitions (*31*–*33*, *52*). Correspondingly, cells in the graph index shift from colorful (finite chirality) to colorless (no chirality). To draw the dashed lines in Fig. 3E, we search for the exceptional points in the Bogoliubov spectrum around every NESS; compare to Fig. 3B. As these commonly precursor a bifurcation, the dashed lines appear around some of the solid transition lines. We denote distinct overdamped regions originating from different spiraling graphs with an additional subscript, e.g., $N_{\alpha ,o}$ . Additional resolution related to the overdamping of specific spirals is mapped to sublabels, detailed further in figs. S2 and S3.

### Saddle connection topological phase transitions

**Fig. 4.**
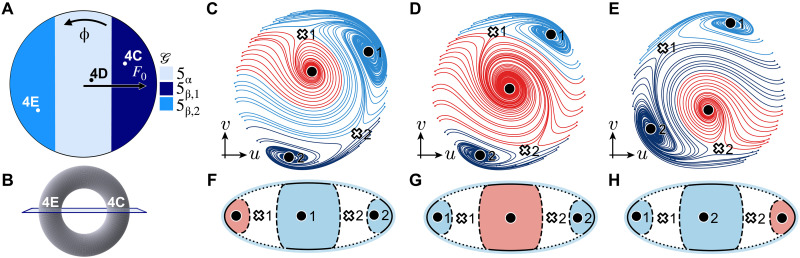
Topological phase transition in vector flow connectivity. (**A**) Schematic phase diagram for the region with *N* = 5 solutions in Fig. 3E, as a function of driving amplitude $F_{0}$ and single-photon driving phase ϕ∈[0,2π] , with fixed two-photon driving phase ψ . (**B**) The transition between $5_{\beta ,1}$ and $5_{\beta ,2}$ via $5_{\alpha }$ resembles a topological ${\mathbb{Z}}_{2}$ transition due to the fixed gauge from the single-phonon drive’s reference phase. This is akin to forcing a transition between two sides of a torus when moving on the cut plane. Figure labels mark positions of other plots. (**C** to **E**) Experimentally measured flows of the phases $5_{\beta ,1}$ , $5_{\alpha }$ , and $5_{\beta ,2}$ , at $F_{0}=500\mathrm{mV}$ , $F_{0}=150\mathrm{mV}$ , and $F_{0}=-500\mathrm{mV}$ , respectively. We keep ω/2π=1.1194MHz constant to highlight the mirror reflection between [(C) and (E)]. Other parameters match those in Fig. 2. (**F** to **H**) Associated graph indexes to the flows [(C) to (E)], respectively.

Last, we switch our focus to the *N* = 5 region in the phase diagram in Fig. 3E, where topologically distinct phases $5_{\alpha }$ and $5_{\beta }$ appear. Here, the vector flow has two clockwise and one counterclockwise sinks, as well as two saddles. The topological transition, here, is driven purely by changes in flow connectivity. At the transition, separatrices connect two saddles, form a heteroclinic link, and thus break structural stability. To better understand the effect, we zoom in on the transition region and vary *F* and ϕ ; see Fig. 4A. We observe three distinct phases. Analogous to crossing a torus along a fixed cut (Fig. 4B), this highlights a ${\mathbb{Z}}_{2}$ distinction between the two π-shifted regions, which we denote $5_{\beta ,1}$ and $5_{\beta ,2}$ . Note that this distinction is possible because of the fixed gauge provided by the drive.

To visualize the different phases, we experimentally reconstruct flows from each of the phases; see Fig. 4 (C to E). The measured flows demonstrate that graph index transitions are caused by a rearrangement in the separatrices in the vector flow. The transition is clearly shown in movie S1, as described in Supplementary Text. At large positive (negative) *F* (Fig. 4, C and E), the flow has a counterclockwise sink that is connected only to saddle 1 (saddle 2). At F≈0 instead (Fig. 4D), both saddles are connected to the counterclockwise sink. We draw the flows’ corresponding graphs in Fig. 4 (F to H) (for additional details, please refer to fig. S4). The graphs defining $5_{\beta ,1}$ and $5_{\beta ,2}$ are related by a ${\mathbb{Z}}_{2}$ symmetry (u,v)↦−(u,v) , which inverts the NESSs through the origin of phase space, a “population inversion.” Note that the fixed gauge in the experiment makes saddles 1 and 2 distinguishable, rendering graphs in Fig. 4 (F and H) nonisomorphic. The ${\mathbb{Z}}_{2}$ transition between these graphs appears as an inversion of the dominant basins of attraction for phonon population in phase space. The latter can serve as an experimental fingerprint for such topological phase transitions. In other words, such a ${\mathbb{Z}}_{2}$ transition will lead to observable changes in noise activation rates between attractors in thermal or quantum regimes (*29*).

## DISCUSSION

In summary, we harness vector field topology to classify the equations of motion arising from the competition between conservative and dissipative forces. We introduce a hierarchy of topological indices that encode the structure of the driven-dissipative flow with increasing resolution. The coarsest level counts the number *N* of fixed points—an integer that tautologically changes only through a (topological) structural instability. A finer level distinguishes fixed points by chirality or damping behavior, tracking how many are clockwise, counterclockwise, or overdamped. The most refined index is a graph invariant based on Morse-Smale theory, capturing how fixed points are connected via separatrices. This full graph encodes not just the type and number of attractors but also their arrangement in phase space. We merge the fixed point chirality into the graphs via colored regions.

Each level of this hierarchy reveals distinct physical phenomena. Changes in *N* mark a change in the number of NESSs in the system and are always visible in thermal or quantum systems that sample all attractors. Changes in chirality correspond to particle- or hole-like excitations and show up in the system’s linear response. Notably, transitions between regions like $3_{\alpha }$ and $3_{\beta }$ involve no change in *N*, but a switch in the relative chirality of the attractors, protected by a multicritical point. Last, transitions where *N* and chirality remain fixed but separatrices rearrange lead to changes in transition pathways and population distributions, as seen in Fig. 4. Our work, thus, elucidates how topology manifests in driven-dissipative nonlinear systems, establishing a theory beyond existing methodologies tailored for linear systems (*14*, *53*). Experimentally, we demonstrate topologically distinct flows in a driven microelectromechanical resonator and reveal a rich emergent phase diagram. For instance, we observe topological population inversion transitions, which would have not been captured with earlier techniques.

Our approach paves the way for the exploration of topological effects in multiresonator systems, including quantum circuits (*46*), many-body collective phenomena in cold atoms (*32*, *33*), nonlinear phonon networks (*54*), and nonlinear optics (*28*). For instance, the topological gap solitons observed in polariton lattices (*55*) align with our classification as isolated, stable modes under coherent drive. It also helps to understand how nonlinearities induce topological phase transitions in photonic topological insulators (*56*) and in Ising machines (*57*). Natural next steps will involve finding a deeper connection between our graph construction and band topology, as well as to extend the classification to limit cycles in similitude to steps taken in (*4*). The classification of the many-body flows in such high-dimensional phase-spaces will rely on extensions of Morse-Smale theory (*9*). An open question is how noise—classical or quantum—affects the flow topology and noise-activated pathways, with implications for neuromorphic computing (*58*), biological information processing (*59*), climate (*60*), machinery fault detection (*61*) sensing (*62*, *63*), and active matter (*64*). Lastly, we expect these topological properties to carry over to driven-dissipative systems deep in the quantum regime, guiding the design of robust quantum phases. They may also enhance quantum bosonic codes like the cat code (*65*), which often rely on the same model and coherent states as our work. Similar ideas could extend to closed quantum systems, drawing on the formal classification of integrable Hamiltonian dynamics (*66*).

## MATERIALS AND METHODS

### Mathematical background: Morse-Smale theory and vector field topology

Formally, a 2D vector field $V:M\to {\mathbb{R}}^{2}$ is a smooth mapping on the manifold M, assigning a vector to each point. A vector flow traces each point’s trajectory over time. Topological analysis allows extracting key features from complex flow patterns, offering a higher-level abstraction of V . This approach helps segment the flow into regions with similar asymptotic behavior. The theory establishes that the main topological features of vector flows are (*5*, *7*, *67*) as follows:

1) Critical points: Locations where the vector field vanishes, including sources, sinks, and saddles. The latter variety is classified through eigenanalysis of the Jacobian matrix related to the local linearization of V around a critical point. The Jacobian determines the flow’s behavior of surrounding trajectories around the critical point.

2) Separatrices: Special trajectories originating or terminating at critical points, forming boundaries between regions of different flow behavior and dividing the flow into distinct asymptotic regions.

3) Periodic orbits: Recurrent flow patterns.

Topological features of vector flows enable the reconstruction of manifold information. In addition, they facilitate the means to establish whether two vector flows V and $V^{\prime }$ on the same manifold M are equivalent. These vector flows are topologically trajectory equivalent if there exists a homeomorphism h:M→M that smoothly maps the trajectories of V to those of $V^{\prime }$ , while preserving their orientations (e.g., their asymptotics) but not necessarily their parameterization (e.g., their speeds). This formal idea can be put into a practical framework through Morse-Smale theory (*4*, *67*).

We introduce Morse-Smale theory through its connection with Morse theory. This topology of topography theory explores the mutual relationship between the topology of M and the distribution of critical points of functions f:M→ℝ defined on it. A Morse function is assumed to have isolated, nondegenerate critical points (where the Hessian determinant is nonvanishing). Morse theory encodes the topology of M in these critical points and their stable/unstable manifolds, which are the sets of points whose trajectories converge to or diverge from the critical points as t→∞ . Note that, near the fixed point, these manifolds are tangent to the eigenspace of the stable eigenvalues of the linearization of ∇f . This is achieved by examining the level sets of a Morse function as they pass through the critical points. Given a fixed manifold M, Morse theory provides a framework to determine the global properties and spatial relationships of features like maxima and minima of the function *f*, focusing on connectivity and relative positions rather than exact shapes or metric distances.

To illustrate an application of Morse theory, consider a mountainous landscape with an elevation function f(x,y) . Rainfall creates watersheds and basins where water accumulates. At low levels, the watersheds are disconnected. Critical points (peaks, valleys, and passes) determine the flow and basin divisions, contour lines represent flow boundaries, and sublevel sets show where water collects. As water levels rise above critical points, watersheds expand and connect, changing their topology, which is independent of the shapes or distances in f . Formally, Morse theory extracts topological information from M using the critical values of *f* and their stable/unstable manifolds. Thus, one calculates the homology group associated to M, which is a topological invariant. The rank of the “free part” of homology groups, known as Betti numbers (*1*), indicates the number of holes in each dimension, with physical implications. In the Aharonov-Bohm effect, for instance, the nontrivial geometric phase accumulation is due to the nontrivial topology of the region outside the solenoid, similar to a cylinder, with a first homology group $H^{1}$ with rank 1 (one noncontractible loop).

A related concept is that of a Morse-Smale dynamical system or flow. In such a flow, (i) there is a finite number of hyperbolic singular points and periodic orbits, (ii) all orbits converge to a singular point or periodic orbit, and (iii) stable and unstable manifolds of singular points and periodic orbits intersect transversely. The gradient of a Morse function, ∇f , induces a Morse-Smale dynamical system when it fulfills the Smale condition: stable and unstable manifolds of the critical points must intersect transversely. Specifically, if x and y are critical points with stable and unstable manifolds $W^{s}\left(x\right)$ and $W^{u}\left(y\right)$ , the transversality condition at any intersection point **w** is $T_{w}\left[W^{s}\left(x\right)\right]+T_{w}\left[W^{u}\left(y\right)\right]=T_{w}\left(M\right)$ , where $T_{w}\left(M\right)$ is the tangent space to M at **w**. This ensures that the stable and unstable manifolds intersect robustly and nondegenerately, unlike when manifolds are tangent. Crucially, in 2D flows, the absence of transversally intersecting stable and unstable manifolds means that there are no separatrices between saddles (*4*).

When the Smale condition holds, there is a direct correspondence between M and the gradient of ∇f . At a critical point of *f*, the gradient flow forms a sink (local minimum), source (local maximum), or saddle point. The separatrices, or “ridge lines,” are boundaries between stable and unstable manifolds: Stable manifolds have trajectories converging to the critical point, while unstable manifolds have trajectories diverging from it. This extends the link between the topology of M and the function *f* to the dynamical system u=−∇f , where u represents coordinates in M. Morse-Smale theory, akin to standard Morse theory for studying the topography of f , provides a framework to extract topological features of vector flows on a manifold M. It focuses on the connectivity and relative positions of critical points rather than their spatial locations.

### Mathematical background: Structural stability and MSCs

A Morse-Smale system has highly structured and well-behaved dynamics, characterized by structural stability, meaning that its qualitative dynamics are preserved under small perturbations (see Introduction). While Morse-Smale systems are structurally stable, the converse holds only for 2D systems, as stated by Peixoto’s theorem (*68*, *69*). Structurally stable vector flows can be described by MSC. The MSC decomposes a manifold M into regions or “crystals” with similar flow behavior around critical points of the vector field. Each critical point corresponds to a cell, and these cells form the building blocks of the complex. The boundaries of the cells are formed by the stable and unstable manifolds of the critical points of a Morse function. The cells in an MSC are connected by gradient lines, which indicate the flow from one critical point to another. In dimension 2, the MSC consists of the following components:

1) Nodes (0-cells): These are the critical points, including minima, saddles, and maxima.

2) Edges (1-cells): These are the separatrices connecting saddles to extrema.

3) 2-cells/Crystals: These are regions bounded by separatrices from saddles. In dimensions larger than 2, the MSC may contain higher-dimensional cells without interior critical points.

The MSC abstracts the topology of a manifold, facilitating efficient computation of its homology groups from the critical points and gradient flow trajectories of a Morse function. A major application of MSCs is in calculating persistent homology in computational topology and data analysis (*7*), which tracks the birth, persistence, and death of topological features, like connected components, loops, and voids, across scales in M. This, e.g., allows efficient feature extraction of from data on a surface, after “filtering out” the key topological information on critical point function values.

MSCs are also crucial for extracting the topological skeleton, or Morse-Smale graph, which captures vector field topology with 0D (vertices) and 1D (edges) cells, highlighting the key features in the trajectory behavior (critical points and separatrices). For 2D flows, this graph can be associated to the so-called Oshemkov-Sharko graph, a topological index detailed in the next section (*4*).

### Mathematical background: Construction of the graph invariant (2D flows)

The Oshemkov-Sharko graph, or three-color graph, is a powerful tool for classifying Morse-Smale flows on compact 2D manifolds (*4*). Namely, two Morse-Smale flows on 2D manifolds are topologically equivalent if and only if their Oshemkov-Sharko graphs are isomorphic, making this graph a complete topological invariant for their classification. Hence, the graph encodes crucial information about the flow, such as the number and types of critical points, their connectivity, and the overall topological structure of the manifold. It provides a combinatorial representation that enables efficient computational analysis, visualization, and classification of Morse-Smale flows. The graph is constructed from the topological skeleton as follows (Fig. 1, C and D): Consider a Morse-Smale gradient flow V on the flat manifold M with at least one saddle point.

We embed the flow on a 2D sphere $S^{2}$ to place it on a compact manifold. This is done by adding the point ∞ and using an inverse stereographic projection $\left(u,v\right)\mapsto \left[2ur^{2},2vr^{2},r\left(A^{2}-r^{2}\right)\right]/\left(A^{2}+r^{2}\right)$ , with $A=\sqrt{u^{2}+v^{2}}$ , on a three-coordinate axis—an example of one-point compactification. A large sphere radius *r* ensures that all critical points are in the southern hemisphere. This compactification is feasible because all flows either emanate from or terminate at infinity, e.g., ensuring that the flow does not rest at any global saddle points. In Fig. 1C, VS is the virtual source at ∞.

The separatrices in the flow split $S^{2}$ into triangles (Fig. 1C) with edges formed by (i) a trajectory from a maximum to a saddle (dotted) and (ii) a trajectory from a saddle to a minimum (dashed). In addition to separatrices, we introduce in the graph construction (iii) a trajectory from a maximum to a minimum (solid). The collection of these dashed lines and the regions they draw on the surface takes the name of MSC. The Oshemkov-Sharko graph ( G ) is the dual of the MSC with the added connections to the VS. Given the aforementioned triangulation and edge dashing, G is a planar graph, constructed as follows:

1) A node in G is added for each triangle.

2) If two triangles are sharing a side in the MSC, the corresponding nodes in G must be connected by a line of the same color.

Consider Fig. 1C. Triangle *a* shares edges with triangles *b* (dotted), *d* (solid), and *e* (dashed). In the planar graph, this translates into node *a* being connected to nodes *b*, *d*, and *e* through a dotted, a solid, and a dashed line, respectively. The collection of all nodes and edges is the Oshemkov-Sharko planar graph Fig. 1D of the vector flow. In building the graph, it is crucial to remember that, as in the MSC, the edges of G cannot cross and that each node of G must be connected to three edges, each of a different dashing. By construction, the resulting graph will have as many regions as the total number of NESS in the vector flow, as shown in Fig. 1D. Each region of G must correspond to one and only one NESS. Since the edge dashing has been preserved in the graph construction, if two regions of G share an edge of a given dashing, then the corresponding NESSs in the vector flow are connected by a separatrix of the same dashing.

The graph construction enables a discrete analog of the Gauss-Bonnet theorem: By deleting all edges of one dashing style, G decomposes into a disjoint union of cycles formed by the remaining two dashing styles. Let $m_{0}\left(G\right)$ , $m_{1}\left(G\right)$ , and $m_{2}\left(G\right)$ denote the number of cycles obtained by deleting the dotted, solid, and dashed edges, respectively. These $m_{i}\left(G\right)$ simply count the number of minima, saddles, and maxima in M. It can be shown [Theorem 1.12 in (*4*)] that the Euler characteristic of the surface M, dubbed χ(M) reads $\chi \left(M\right)=m_{0}\left(G\right)-m_{1}\left(G\right)+m_{2}\left(G\right)$ (an example of the so-called Poincaré-Hopf theorem).

In the present study, the only maximum corresponds to an artificial or “virtual” source (the added ∞ point at the north pole of the sphere), introduced to account for all flow lines coming from infinity when embedding the flow on a sphere (Fig. 3, C and D, and fig. S4, top row). As described in the Results, Topology of nonequilibrium vector flows section, our goal is to encode chirality information directly into the planar graph. To this end, we assign colors to regions of the graph based on the chirality of the trajectories around the corresponding attractor (blue for clockwise and red for counterclockwise). The graph’s skeleton is constructed using the Oshemkov-Sharko procedure outlined above. We then identify the attractor regions and color them according to their chirality. Since the flow evolves in a 2D rotating phase space, we also add a surrounding halo, uniformly colored by the chirality of the asymptotic background flow. Together, this construction yields a graph invariant that encodes not only the number and connectivity of critical points but also their chirality (Fig. 3, C and D, and fig. S4, bottom row). Thus, our topological classification encompasses gap-closing transitions in the Bogoliubov–de Gennes Hamiltonian around every NESS in the system.

### Experimental setup

Our MEMS device (*38*, *39*) consists of a mechanical tuning fork, placed in between two electrodes for capacitive driving and readout. The MEMS device, as discussed in (*41*), is biased with a static voltage *U*_bias_ = 32 V via one electrode and driven by applying oscillating voltages corresponding to a single-phonon drive (voltage amplitude, $F_{0}$ ; frequency, ω ) and/or a two-phonon drive (voltage amplitude, $G_{0}$ ; frequency, 2ω ). The mechanical motion $x\propto U_{\text{out}}$ is read out from the voltage $U_{\text{out}}$ at the other electrode. Quadratures *u* and *v* are extracted via a lock-in amplifier operating at a frequency ω.

### Experiment: Device parameters

For simplicity, we treat the device as an effective electrical resonator (*40*) with “displacement” ζ (in units of V), which we directly measure and that can be cast into an equation of motion as

$$\ddot{\zeta }+\omega_{0}^{2}\left[1+\lambda \text{cos}\left(2\omega t\right)\right]\zeta +\gamma \dot{\zeta }+k_{3}\zeta^{3}=\tilde{f}\text{cos}\left(\omega t+\phi \right)$$ (5)

whose parameters are directly extracted from measurements (*41*). We find a natural frequency $\omega_{0}/\left(2\pi \right)\approx 1.1198\mathrm{MHz}$ , a Duffing nonlinearity $k_{3}\approx -9.9\times 10^{16}V^{-2}s^{-2}$ , and an energy decay rate γ/(2π)≈112Hz . The single-phonon drive voltage $F_{0}$ is related to $\tilde{f}$ (with units of ${\mathrm{Vs}}^{-2}$ ) as $\tilde{f}=\eta F_{0}$ , with $\eta \approx 1.6\times 10^{7}s^{-2}$ . Here, the resonator’s effective mass is absorbed into $\tilde{f}$ . The two-phonon drive voltage amplitude $G_{0}$ can be converted to a unitless parametric drive modulation strength via $\lambda =\nu G_{0}$ with $\nu \approx 2\gamma /\left(\omega_{0}U_{\text{th}}\right)\approx 10^{-4}V^{-1}$ , where *U*_th_ ≈ 1.98 V marks the lower driving threshold for parametric oscillation (*31*). Since voltage displacement ζ and mechanical displacement *x* are proportional, we will express our theoretical model in the following using *x* for generality.

### Experiment: Stationary amplitude measurement

To measure the stationary amplitude of the device under both single- and two-phonon drives (compare to Fig. 2B), we apply a two-phonon drive with amplitude *G*_0_ = 3.2 V at frequency 2ω , and a single-phonon drive of phase ϕ=0.47π with varying amplitude $F_{0}$ at frequency ω . For each fixed single-phonon drive amplitude $F_{0}$ , we sweep ω from low to high frequencies while recording the lock-in amplifier’s demodulated quadratures *u* and *v* at frequency ω , and calculate the stationary amplitude $A=\sqrt{u^{2}+v^{2}}$ . The waiting time at each point in the stability diagram is much longer than $\gamma^{-1}$ to avoid measuring transient effects.

### Experiment: Vector flow measurement

To measure the vector flow dynamics (e.g., Fig. 2C), we use a similar method as the one described in (*41*). We first displace the mechanical element in the rotating-frame phase space, spanned by q=(u,v) , by applying a near-resonant drive ( $\omega \approx \omega_{0}$ ). The phase and amplitude of this drive set the initial condition for a single-vector flow measurement and is marked as single dots in the flow measurements figures. This initialization drive is then switched off, and both a single-phonon drive at frequency ω and a two-phonon drive at frequency 2ω are immediately switched on, while recording the lock-in amplifier demodulated quadratures *u*(*t*) and *v*(*t*) at frequency ω . The oscillator “rings down” in the rotating quasi-potential created by these two drives, which yields a single trace in the rotating phase-space (*41*). This measurement is repeated for varied initial conditions, allowing us to sample the oscillator’s dynamics over a large area of phase-space and map out the vector flow dynamics. The frequency ω , the near-resonant single-phonon drive’s phase ϕ and amplitude $F_{0}$ , as well as the parametric two-phonon drive amplitude $G_{0}$ are all experimentally controllable parameters and allow us to tune the quasi-Hamiltonian of the system and explore various topological phases.

## Supplementary Materials

### The PDF file includes:

Supplementary Text Figs. S1 to S4 Legend for movie S1 References
