# Supplementary material for: Topological classification of driven-dissipative nonlinear systems
Source: Sci Adv. 2025 Aug 13;11(33):eadt9311. doi: 10.1126/sciadv.adt9311 (PMC12346257; doi:10.1126/sciadv.adt9311)
Supplement: Supplementary file 1 — Supplementary Text Figs. S1 to S4 Legend for movie S1 References [file sciadv.adt9311_sm.pdf]

Science Advances
AAAS

Supplementary Materials for

Topological classification of driven-dissipative nonlinear systems

Greta Villa *et al.*

Corresponding author: Javier del Pino, j.delpino@uam.es;
Oded Zilberberg, oded.zilberberg@uni-konstanz.de

Sci. Adv. **11**, eadt9311 (2025)
DOI: 10.1126/sciadv.adt9311

The PDF file includes:

Supplementary Text
Figs. S1 to S4
Legend for movie S1
References

Other Supplementary Material for this manuscript includes the following:

Movie S1

Supplementary Text

Effective Hamiltonian derivation

We use a Floquet framework to model nonlinear resonator dynamics under parametric and resonant driving. The starting point is the Hamiltonian in the lab frame for an oscillator with amplitude operator x and momentum operator p:

$$H = \frac{1}{2}\left(\frac{p^2}{m} + m\tilde{\omega}_0(t)^2 x^2\right) + m\frac{k_3}{4}x^4 - \tilde{F}\cos(\omega_d t + \phi)x\,. \tag{S1}$$

Here, m is the oscillator's mass, k_3 represents the Kerr nonlinearity, $\tilde{\omega}_0(t) = \omega_0(1 - \lambda\cos(\omega_p t + \psi))$ is a time-dependent frequency, oscillating around the natural frequency ω_0, with modulation frequency ω_p, depth λ, and phase ψ. $\tilde{F}$ quantifies the amplitude of an external periodic force with frequency ω_d and phase ϕ. We use the symbols x and ζ interchangeably since $x \propto \zeta$. Parameters in Eq. S1 are related to the voltage signal evolution in Eq. 5 by the voltage-to-mechanical-displacement conversion factors, which are left implicit. Therefore we use the same symbols in both equations, noticing that $\tilde{F} = \tilde{f}m$. The Hamiltonian in the linear, unforced limit ($k_3 = \tilde{F} \equiv 0$), generates the Mathieu equation (*70, 71*) as the equation of motion for x, whose solutions are characterized by an infinite hierarchy of high harmonics. The phase diagram of the Mathieu equation as a function of the parameters λ and ω_0/ω_p shows regions with unbounded oscillations, or "Arnold tongues", indicating parametric resonance at values of the parametric driving frequency whenever $\omega_0/\omega_p \approx n$, with $n \in \mathbb{N}$. When $\lambda = 0$ and $k_3 \neq 0$, the system behaves as a driven Duffing oscillator. It can exhibit bistable oscillation regions, with steady states of low and high amplitude corresponding to different fixed points in the dynamical equations (*31*).

We examine the steady state of the oscillator in Eq. (S1) with a negative Kerr parameter ($k_3 < 0$) around the first Arnold tongue, and when fixing $\omega_d = \omega = \omega_p/2$. A similar analysis applies for a positive Kerr nonlinearity. The Hamiltonian (S1) is commonly studied in quantum engineering contexts, including nanomechanics (*30*), driven-dissipative atomic ensembles (*43*) and superconducting cavities (*65*). We quantize the Hamiltonian using canonical operators that count the number of drive phonons, by redefining quantum operators in a basis that matches the driving frequency (*72*). This reconciles the classical and Heisenberg equations of motion after the application of a rotating wave approximation in a rotating frame at the driving frequency ω, even when non-negligible detuning from resonance ($\omega \neq \omega_0$) is present (*46*). Specifically, we define

creation and annihilation operators $c^\dagger$ and c such that

$$x = \sqrt{\frac{\hbar}{2m\omega}}(c^\dagger + c), \qquad p = i\sqrt{\frac{\hbar m\omega}{2}}(c^\dagger - c). \tag{S2}$$

We now switch to a rotating frame at frequency ω, following signal processing and lock-in amplifier conventions $\langle x\rangle = u\cos(\omega t) - v\sin(\omega t)$ with quadratures u, v. We thus define rotating annihilation operators as

$$b = e^{i\omega t}c \equiv \sqrt{m\omega/(2\hbar)}(X + iY)\,, \tag{S3}$$

and $b^\dagger = \sqrt{m\omega/(2\hbar)}(X - iY) =$, where X and Y are canonically conjugate quadratures that fulfil the commutation relation $[X, Y] = 2i\hbar/(m\omega)$ with classical averages $\langle X\rangle = u$ and $\langle Y\rangle = -v$. Note that under this choice, $c = e^{i\omega t}b$, $\langle x\rangle = \langle X\rangle\cos(\omega t) + \langle\mathcal{V}\rangle\sin(\omega t) = u\cos(\omega t) - v\sin(\omega t)$. This choice of quadratures is natural during canonical quantization using a Lagrangian formalism with an ansatz $x_{\mathrm{cl}} = u\cos(\omega t) - v\sin(\omega t)$ transformed into a Hamiltonian via the Legendre transformation, as it recovers the classical Poisson brackets $\{u, -v\} = 2/\omega$. This differs from quantum optics and cavity optomechanics, where quadratures are derived from rotation around the resonance frequency ω_0, resulting in positive Poisson brackets and standard harmonic oscillator quantization (*73*).

Inserting the expressions for b and $b^\dagger$ into Eq. (S1), we find that certain terms in the Hamiltonian oscillate rapidly compared to ω. To achieve a time-independent description, we use a Floquet expansion with van Vleck's degenerate perturbation theory. To first order in $1/2\omega$, this approximation is equivalent to the Rotating Wave Approximation, and reduces Eq. S1 to

$$H/\hbar \approx (-\Delta + U)\, b^\dagger b + \frac{U}{2}b^{\dagger 2}b^2 - \frac{G}{2}\left(b^2 e^{i\psi} + b^{\dagger 2}e^{-i\psi}\right) - F\left(be^{i\phi} + b^\dagger e^{-i\phi}\right)\,, \tag{S4}$$

where $\Delta = (\omega^2 - \omega_0^2)/2\omega$, $U = 3\hbar k_3/(4m\omega^2)$, $G = \omega_0^2\lambda/(2m\omega)$, $F = \tilde{F}/(2\sqrt{2m\omega\hbar})$. The effective detuning Δ, nonlinearity U, two phonon drive G, and pump strength F are assumed to be small compared with $\hbar\omega$. Henceforth, we fix the parametric phase at $\psi = 0$ and scale units such that $m = 1$.

Semiclassical, open quantum system evolution

Besides the external driving, the resonator is unavoidably coupled to its environment, leading to damping. To describe the open quantum system evolution in the Markovian limit, we introduce

a Lindblad superoperator for b, modelling interaction with a memoryless (Markovian) oscillator environment. The master equation for the reduced density matrix ρ reads:

$$\frac{d\rho}{dt} = -\frac{i}{\hbar}[H, \rho] + \mathcal{L}[\rho], \tag{S5}$$

where H follows from Eq. (S4), $\mathcal{L}[\rho] = \gamma\left(b\rho b^\dagger - \frac{1}{2}\{b^\dagger b, \rho\}\right)$ represents the action of the Lindblad superoperator on ρ, γ is the loss rate, and $\{A, B\} = AB + BA$ denotes the anticommutator (*31*). This theory assumes that the environment is in a zero-temperature vacuum state. Unlike the usual Lindblad master equation using annihilation operators that count phonons, the vacuum state may be a non-empty, squeezed vacuum (*72*) (*46*). These effects could impact the accuracy of the Lindblad equation with strong system-environment coupling, but we defer this investigation to future work.

From the Lindblad master equation in Eq. (S5), we derive the equations of motion of the average of the operator b, by noting $\langle b\rangle = \mathrm{Tr}(\rho b)$, namely $\frac{d}{dt}\langle b\rangle = \langle[b, H]\rangle/\hbar + \langle\frac{\partial b}{\partial t}\rangle$. In the classical limit $\hbar \to 0$, we obtain

$$i\frac{d\langle b\rangle}{dt} = (-\Delta - i\frac{\gamma}{2} + U|\langle b\rangle|^2)\langle b\rangle - Ge^{-i\psi}\langle b\rangle^* - Fe^{-i\phi}. \tag{S6}$$

We extract the equations for u and v from the real and imaginary parts of this equation. For notational simplicity, we omit the brackets, yielding (gauge $\psi = 0$):

$$\dot{u} = -\frac{\gamma u}{2} + \frac{v(\omega^2 - \omega_0^2)}{2\omega} - \frac{3k_3A^2v}{8\omega} - \frac{v\lambda\omega_0^2}{4\omega} + \frac{f\sin(\phi)}{2\omega}, \tag{S7a}$$

$$\dot{v} = -\frac{\gamma v}{2} - \frac{u(\omega^2 - \omega_0^2)}{2\omega} + \frac{3k_3A^2u}{8\omega} - \frac{u\lambda\omega_0^2}{4\omega} - \frac{f\cos(\phi)}{2\omega}. \tag{S7b}$$

where $A = \sqrt{u^2 + v^2}$ is the resonator amplitude at frequency ω. The equations (S7) can be derived using the classical Krylov-Bogolibov formalism (*30, 31*).

Steady state analysis: dimensional reduction

When working with systems like Eq. (S7), it is useful to map out all steady states as they vary with parameters. However, the number of tunable parameters can exceed the visualization capabilities of phase diagrams, providing only a partial view of the system's behavior. Here, we employ a dimensional reduction technique to reduce the number of parameters by one.

Let us consider the dimensionless equations of motion in Eq. (S7) and solve for the steady states $(\dot{u}, \dot{v}) = (0, 0)$. We introduce dimensionless variables (*29*, *74*):

$$\bar{u} = u\sqrt{3|k_3|/(2\lambda\omega_0^2)}, \qquad \bar{v} = v\sqrt{3|k_3|/(2\lambda\omega_0^2)}. \tag{S8}$$

Next, we introduce the symbol $\Delta\Omega = (\bar{\Omega}^2 - 1)/\bar{\Omega}$ with dimensionless detuning $\bar{\Omega} = \omega/\omega_0$ and divide both equations of motion by a reduced frequency $\xi = \lambda^2/(6\bar{\Omega})$. This allows us to group the parameters into a comprehensive force term $\Lambda = \sqrt{6\bar{f}^2/\lambda^3}$, where the dimensionless force amplitude reads $\bar{\tilde{F}} = \tilde{F}\sqrt{|k_3|/\omega_0^3}$, which encapsulates both driving contributions. Another key parameter is the threshold detuning $\Delta\bar{\Omega}_T = \lambda/(4\bar{\Omega})$, indicating when self-sustained oscillations at $\bar{\Omega}$ begin (in the $\bar{\tilde{F}} = 0$ and $\gamma = 0$ limits). The rescaled steady conditions read:

$$0 = -\bar{v} + \bar{v}^3 + \bar{v}\bar{u}^2 + \frac{\bar{v}\Delta}{2} - \Gamma\bar{u} + \Lambda\sin(\phi), \tag{S9a}$$

$$0 = -\bar{u} - \bar{u}^3 - \bar{u}\bar{v}^2 - \frac{\bar{u}\Delta}{2} - \Gamma\bar{v} - \Lambda\cos(\phi), \tag{S9b}$$

with a damping-related factor $\Gamma = \gamma/(2\Delta\omega_0\bar{\Omega}_T)$. Eqs. (S9) can be recast into a single quintic equation for the squared amplitude $\bar{A}^2 = \bar{v}^2 + \bar{u}^2$:

$$\bar{A}^2\left[4(-1+\Gamma^2) + (2\bar{A}^2+\Delta)^2\right]^2 = 4\Lambda^2\left[4(1+\Gamma^2) + (2\bar{A}^2+\Delta)^2 - 4(2\bar{A}^2+\Delta)\cos(2\phi) + 8\Gamma\sin(2\phi)\right]. \tag{S10}$$

Drawing from the polynomial above, we can derive an analytical expression for the bifurcation surfaces as functions of three parameters. By computing the discriminant of the polynomial in Eq. (S10), we can determine properties of the polynomial roots in terms of Λ, Δ, and Γ without explicit computation. The discriminant of a quintic polynomial, which is zero if and only if the polynomial has a repeated root, determines whether the polynomial has 1, 3, or 5 real roots. For clarity, these expressions — a high degree polynomial– are omitted here. The bifurcation surfaces are accessed by evaluating the roots of the discriminant for each of the three rescaled parameters. Notably, for a closed system where $\Gamma = 0$, this procedure analytically yields the bifurcation lines of the phase diagram.

Extraction of topological flow features: Numerical considerations

Critical points of a function are where its gradient vanishes. If the vector flow is polynomial, finding these points involves solving the real roots or a nonlinear system of algebraic equations, which can

be accomplished via, e.g., Homotopy Continuation (75, 76). After identifying critical points where the vector field vanishes, we linearize the vector field around each critical point to determine its type (source, sink, saddle, etc.).

Separatrices are the fastest trajectories connecting critical points of different curvatures, calculated by tracking forward and backward time evolution from near an unstable singular point. In a gradient-like flow, maxima (sources) have only exiting flow lines, minima (sinks) have only entering flow lines, and saddles have two entering and two exiting flow lines. From saddle points, the separatrices are found by solving differential equations with initial conditions near the saddle point. Using numerical integration techniques like Runge-Kutta methods (77), we compute trajectories starting from points near these singular points (e.g. by displacing from a saddle point and computing trajectory evolutions for all angles). An analogous procedure can be carried out for the source.

Phase diagram in the Kerr Parametron and Kerr driven limits

Here, we discuss the system's phase diagram in the Kerr parametron limit (KPO, $F = 0$) and the driven Kerr limit (KDO, $G = 0$) [see Fig. S1]. In the KPO limit, the phase diagram shows a first Arnold tongue at $\omega = \omega_0/2$, with up to five stationary states. Depending on parameters, the number of stationary states ranges from one (green region) to three (burgundy region) to five (blue region). Parametric resonance occurs for negative detunings due to the resonator's negative dispersive shift with a softening nonlinearity ($k_3 < 0$). Sweeping the frequency across the parametric resonance (at $\bar{\Omega} = 1$) with fixed modulation strength λ, we traverse all regions. Starting from the green region, the only solution is a zero-amplitude steady state ($u = v = 0$), or "normal phase". Transitioning from this single-solution state to the three-solution region involves a supercritical pitchfork bifurcation. Here, the stable zero-amplitude state becomes unstable and splits into two stable equal-amplitude states, related by a broken $\mathbb{Z}_2$ symmetry $(u, v) \rightarrow -(u, v)$. This transition resembles a second-order phase transition. Further sweeping leads to a transition from the burgundy to the blue region via a subcritical pitchfork bifurcation, where the unstable solution generates a stable zero-amplitude state and two unstable states, leading to the coexistence of phase states with a zero-amplitude state in the blue region.

In the KDO limit, a similar analysis reveals up to three solutions. During a frequency up-sweep with fixed $F > 0$, the system traverses regions with one (green) and three (pink) solutions, of which

one solution and two are stable, respectively. The green normal phase region, corresponds to a displaced, detuned harmonic oscillator solution. In the bistable region, a low-density "gas" phase coexists with a high-density "liquid" phase. The separation between the two regions, marked by a saddle-node bifurcation, resembles a first-order phase transition (*31, 43*).

We apply the classification scheme related to overdamping, cf. Fig. S1, S2 and S3 for further information. In Fig. S1**A** and **C**, we show the phase the KPO and KDO limits, respectively, using N as the only classifier. In Fig. S1**B** and **D**, we present the same phase diagram, applying vector flow analysis. While N can only distinguish between three values (Fig. S1**E**, column on the left), vector flow analysis offers a much a complete and exhaustive description of the system (Fig. S1**E**, column on the right). The same is performed in Fig. S2, showing that N is also blind to process where separatrices rearrange. Indeed, the complete classification we showcase in Fig. S3 uncovers a plethora of phases within the phase diagram.

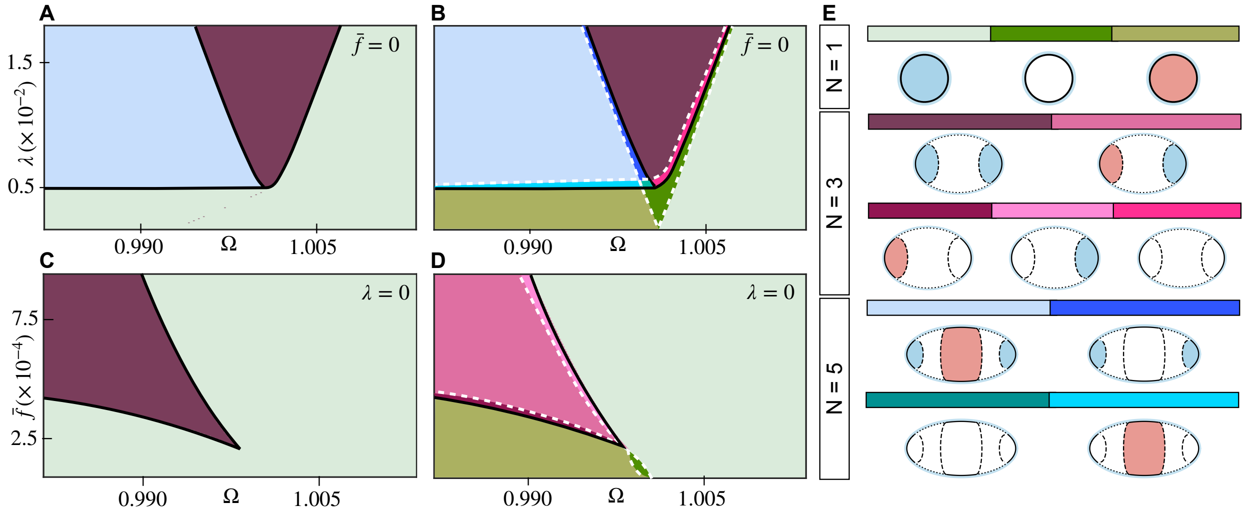

Figure S1: **Phase Diagram in the Parametron and Kerr Duffing Limits a.** Number of solutions phase diagram for a pure Kerr Parametric oscillator. Green, wine, and blue indicate regions with $N = 1$, $N = 3$, and $N = 5$ critical points, respectively. Here, we parametrized the two-phonon driving strength using modulation λ, related to the Hamiltonian parameter G as $\lambda = \frac{4G\omega}{\omega_0^2}$ (see Supplementary Text). **b.** Subclassification based on our topological graph framework, showing further region subdivisions with distinct colors. **c.** and **d.** are the analogs of **a.** and **b.** in the Kerr Duffing Oscillator limit. **e.** Correspondence table of colors with graph invariants. Crucially, here we observe that an overdamped region is accessible also when $N = 1$.

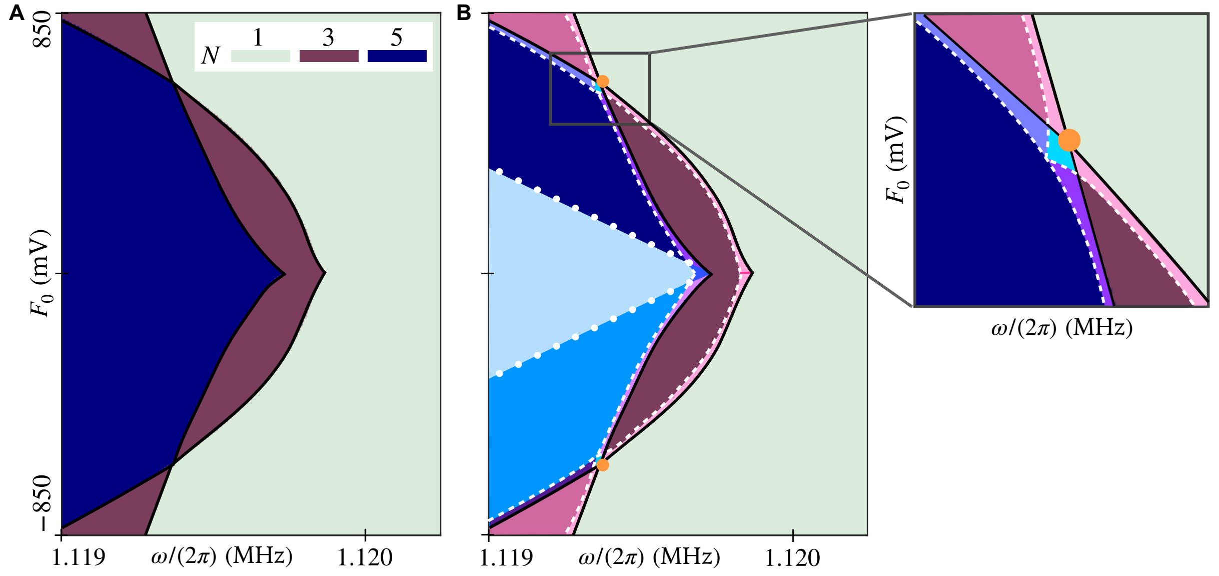

Figure S2: **System's full phase diagram. a.** Phase diagram based on solutions count. The upper half ($F_0 \geq 0$) reconstructs Fig. 2b or Fig. 3e. **b.** Vector flow analysis reveals further sub-regions in the phase diagram with three phase transition types: changing N (solid), under-to-overdamped (dashed), and separatrix reconnection (dotted). Overdamped regions arise when spirals turn into non-chiral sinks. Enhancing the graph invariant by distinguishing sinks that become overdamped and the chiralities of the remaining ones reveals various phases in the $N = 5$ region: ↺ (CCW), ↻ (CW), and × (overdamped). We organize these labels in a triad for the three sinks, ordered as (↺, ↻$_1$, ↻$_2$), where the subindex (1,2) differentiates between the two CW sinks (see Fig. S1 legend). Chirality loss occurs near transitions to fewer solutions as a sink merges with a saddle. As in Fig. 3, the spacing between dashed and solid lines is magnified for clarity. **Inset:** Zoom in around the multicritical point; the overdamped region of 5_β shows three possible invariants, depending on which sink loses chirality: a ↻ (violet), a ↺ (purple), or both (bright light blue). $5_{\beta,o}$ is close to the multicritical point, where the $N = 5 \longrightarrow N = 1$ phase transition occurs. Two sink-saddle pairs (one containing a CW sink, one containing a CCW sink) merge, leaving one ↻ sink retaining chirality.

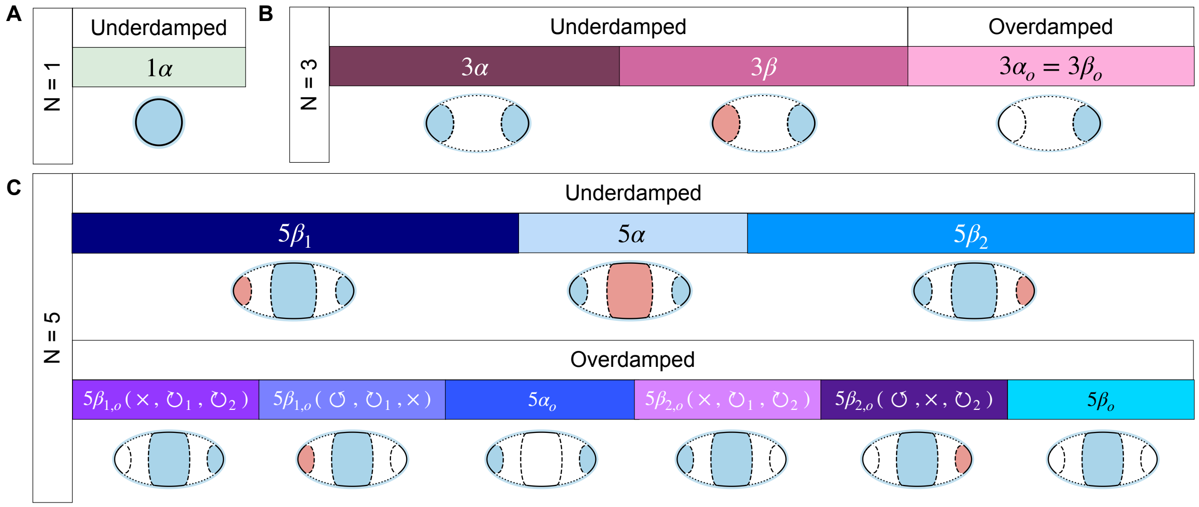

Figure S3: **Graph invariant Catalogue of System Properties.** Table containing all possible graph invariants in the system, constructed from vector flows corresponding to different regions in Extended Data Fig. S1: **A** single solution, **B** 3 solutions, and **C** 5 solutions. Heading colors match the phase diagram region colors. $3_{\alpha,o} = 3_{\beta,o}$, as they both transit in the same $N = 1$ region.

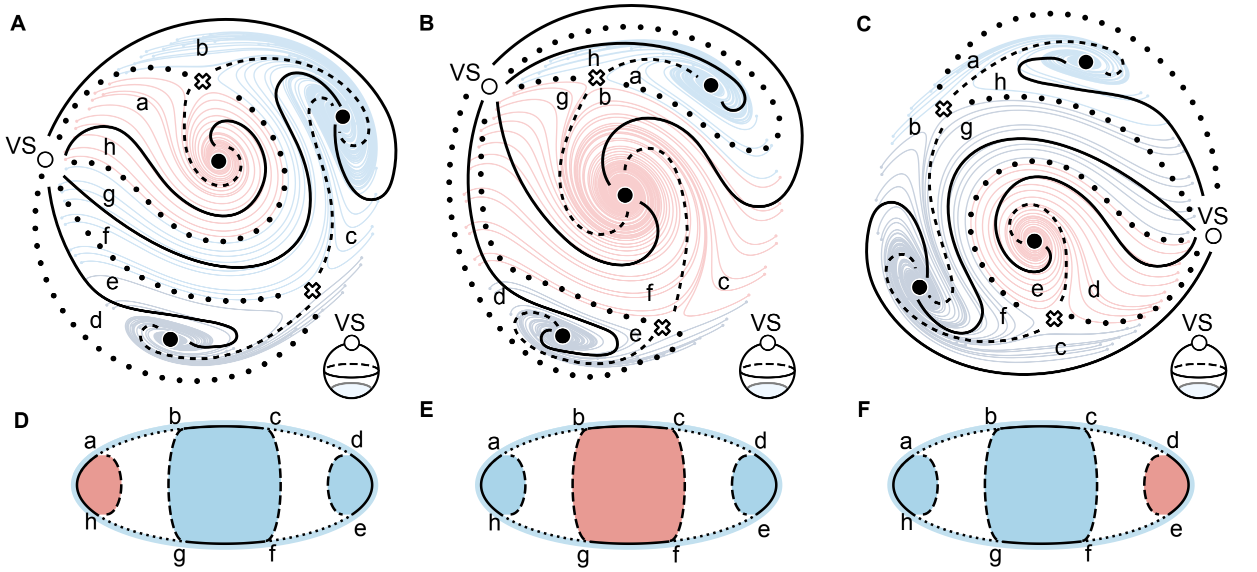

Figure S4: **Construction of the graph invariants in the** $N = 5$ **regions.** Morse–Smale graphs on the sphere. The virtual source (VS) is added via one-point compactification (inset). Curves topologically equivalent to separatrices of the flow are displayed, with dashing marking their type: dotted (VS–saddle), dashed (saddle–sink), solid (VS–sink). Regions bound by the separatrices are labeled with lowercase letters. **A**, **B**, **C** correspond to topological phases $5_{\beta,1}$, 5_{α} and $5_{\beta,2}$ respectively. The corresponding graph indexes are shown in **D**, **E** and **F**. Here graph index nodes are labelled by corresponding letters to the regions in **A**, **B** and **C**, and the curves connecting them reflect the dashing and arrangement of the separatrices in the MSC. Panels **A,D**, panels **B,E**, and **C,F** show the flows and graphs from Fig. 4**C**, **D**, and **E** in the main text, respectively.

Caption for Movie S1. Separatrix rearrangement transition. Movie S1 illustrates the mechanism behind the transition reported in main text Fig. 4. In the flow, there is a counter-clockwise (CCW) sink and two clockwise (CW) sinks. For a large negative single photon drive (denoted as $-F_m$), separatrices connect the CCW sink with one of the saddles. Each saddle is a point where four separatrices cross (two incoming and two outgoing). Around $F = 0$, the CCW sink is connected to the two existing saddles. As F becomes large positive (maximum denoted F_m), separatrices in the flow only connect the CCW sink with the opposite saddle.

During these separatrix reconnections, the number of solutions and their character (saddle and sink chirality) remain fixed, highlighting the nonlocal nature of this topological phase transition. This is reflected by the changes in the graph invariant reported in the main text.

REFERENCES AND NOTES

1. A. Hatcher, *Algebraic Topology* (Cambridge Univ. Press, 2002).

2. M. F. Atiyah, *The Geometry and Physics of Knots* (Cambridge Univ. Press, 1990).

3. A. A. Andronov, L. S. Pontryagin, Systèmes grossiers. *Dokl. Akad. Nauk SSSR* **14**, 247–250 (1937).

4. A. A. Oshemkov, V. V. Sharko, Classification of Morse-Smale flows on two-dimensional manifolds. *Sb. Math.* **189**, 1205 (1998).

5. M. Morse, Topology and equilibria. *Am. Math. Mon.* **114**, 819–834 (2007).

6. J. J. Palis, W. De Melo, *Geometric Theory of Dynamical Systems: An Introduction* (Springer Science & Business Media, 2012).

7. T. Günther, I. Baeza Rojo, "Introduction to vector field topology," in *Topological Methods in Data Analysis and Visualization VI: Theory, Applications, and Software* (Springer, 2021), pp. 289–326.

8. D. W. Longcope, Topological methods for the analysis of solar magnetic fields. *Living Rev. Sol. Phys.* **2**, 7 (2005).

9. G. Carlsson, Topological methods for data modelling. *Nat. Rev. Phys.* **2**, 697–708 (2020).

10. R. Gilmore, Topological analysis of chaotic dynamical systems. *Rev. Mod. Phys.* **70**, 1455–1529 (1998).

11. R. Gilmore, M. Lefranc, *The Topology of Chaos: Alice in Stretch and Squeezeland* (John Wiley & Sons, 2012).

12. M. Z. Hasan, C. L. Kane, Colloquium: Topological insulators. *Rev. Mod. Phys.* **82**, 3045–3067 (2010).

13. B. A. Bernevig, *Topological Insulators and Topological Superconductors* (Princeton Univ. Press, 2013).

14. T. Ozawa, H. M. Price, A. Amo, N. Goldman, M. Hafezi, L. Lu, M. C. Rechtsman, D. Schuster, J. Simon, O. Zilberberg, I. Carusotto, Topological photonics. *Rev. Mod. Phys.* **91**, 015006 (2019).

15. T. Shah, C. Brendel, V. Peano, F. Marquardt, Colloquium: Topologically protected transport in engineered mechanical systems. *Rev. Mod. Phys.* **96**, 021002 (2024).

16. N. R. Cooper, J. Dalibard, I. B. Spielman, Topological bands for ultracold atoms. *Rev. Mod. Phys.* **91**, 015005 (2019).

17. E. J. Bergholtz, J. C. Budich, F. K. Kunst, Exceptional topology of non-Hermitian systems. *Rev. Mod. Phys.* **93**, 015005 (2021).

18. J. J. Slim, C. C. Wanjura, M. Brunelli, J. del Pino, A. Nunnenkamp, E. Verhagen, Optomechanical realization of the bosonic Kitaev chain. *Nature* **627**, 767–771 (2024).

19. E. Fradkin, *Field Theories of Condensed Matter Physics* (Cambridge Univ. Press, 2013).

20. S. Rachel, Interacting topological insulators: A review. *Rep. Prog. Phys.* **81**, 116501 (2018).

21. A. Altland, B. D. Simons, *Condensed Matter Field Theory* (Cambridge Univ. Press, 2010).

22. J. L. Lado, O. Zilberberg, Topological spin excitations in Harper-Heisenberg spin chains. *Phys. Rev. Res.* **1**, 033009 (2019).

23. S. Xia, D. Jukić, N. Wang, D. Smirnova, L. Smirnov, L. Tang, D. Song, A. Szameit, D. Leykam, J. Xu, Z. Chen, H. Buljan, Nontrivial coupling of light into a defect: The interplay of nonlinearity and topology. *Light Sci Appl* **9**, 147 (2020).

24. S. Mukherjee, M. C. Rechtsman, Observation of Floquet solitons in a topological bandgap. *Science* **368**, 856–859 (2020).

25. S. Mittal, G. Moille, K. Srinivasan, Y. K. Chembo, M. Hafezi, Topological frequency combs and nested temporal solitons. *Nat. Phys.* **17**, 1169–1176 (2021).

26. N. Mostaan, F. Grusdt, N. Goldman, Quantized topological pumping of solitons in nonlinear photonics and ultracold atomic mixtures. *Nat. Commun.* **13**, 5997 (2022).

27. B. Apffel, R. Fleury, Experimental observation of topological transition in linear and nonlinear parametric oscillators. *Phys. Rev. E* **109**, 054204 (2024).

28. Y.-R. Shen, *Principles of Nonlinear Optics* (Wiley-Interscience, 1984).

29. M. Dykman, *Fluctuating Nonlinear Oscillators: From Nanomechanics to Quantum Superconducting Circuits* (Oxford Univ. Press, 2012).

30. A. Leuch, L. Papariello, O. Zilberberg, C. L. Degen, R. Chitra, A. Eichler, Parametric symmetry breaking in a nonlinear resonator. *Phys. Rev. Lett.* **117**, 214101 (2016).

31. A. Eichler, O. Zilberberg, *Classical and Quantum Parametric Phenomena.* (Oxford Univ. Press, 2023).

32. M. Soriente, T. L. Heugel, K. Omiya, R. Chitra, O. Zilberberg, Distinctive class of dissipation-induced phase transitions and their universal characteristics. *Phys. Rev. Res.* **3**, 023100 (2021).

33. F. Ferri, R. Rosa-Medina, F. Finger, N. Dogra, M. Soriente, O. Zilberberg, T. Donner, T. Esslinger, Emerging dissipative phases in a superradiant quantum gas with tunable decay. *Phys. Rev. X* **11**, 041046 (2021).

34. F. Mivehvar, F. Piazza, T. Donner, H. Ritsch, Cavity QED with quantum gases: New paradigms in many-body physics. *Adv. Phys.* **70**, 1–153 (2021).

35. M. J. Hartmann, F. G. S. L. Brandao, M. B. Plenio, Quantum many-body phenomena in coupled cavity arrays. *Laser Photonics Rev.* **2**, 527–556 (2008).

36. H. Ritsch, P. Domokos, F. Brennecke, T. Esslinger, Cold atoms in cavity-generated dynamical optical potentials. *Rev. Mod. Phys.* **85**, 553–601 (2013).

37. G. W. Wolf, Surfaces–topography and topology. *Surf Topogr Metrol Prop* **8**, 014003 (2020).

38. M. Agarwal, S. A. Chandorkar, H. Mehta, R. N. Candler, B. Kim, M. A. Hopcroft, R. Melamud, C. M. Jha, G. Bahl, G. Yama, T. W. Kenny, B. Murmann, A study of electrostatic force nonlinearities in resonant microstructures. *Appl. Phys. Lett.* **92**, 104106 (2008).

39. P. M. Polunin, Y. Yang, M. I. Dykman, T. W. Kenny, S. W. Shaw, Characterization of MEMS resonator nonlinearities using the ringdown response. *J. Microelectromechanical Syst.* **25**, 297–303 (2016).

40.. Nosan, P. Märki, N. Hauff, C. Knaut, A. Eichler, Gate-controlled phase switching in a parametron. *Phys. Rev. E* **99**, 062205 (2019).

41. V. Dumont, M. Bestler, L. Catalini, G. Margiani, O. Zilberberg, A. Eichler, Energy landscape and flow dynamics measurements of driven-dissipative systems. *Phys. Rev. Res.* **6**, 043012 (2024).

42. C. C. Wanjura, J. J. Slim, J. del Pino, M. Brunelli, E. Verhagen, A. Nunnenkamp, Quadrature nonreciprocity in bosonic networks without breaking time-reversal symmetry. *Nat. Phys.* **19**, 1429–1436 (2023).

43. N. Bartolo, F. Minganti, W. Casteels, C. Ciuti, Exact steady state of a Kerr resonator with one- and two-photon driving and dissipation: Controllable Wigner-function multimodality and dissipative phase transitions. *Phys. Rev. A* **94**, 033841 (2016).

44. T. L. Heugel, M. Biondi, O. Zilberberg, R. Chitra, Quantum transducer using a parametric driven-dissipative phase transition. *Phys. Rev. Lett.* **123**, 173601 (2019).

45. G. Beaulieu, F. Minganti, S. Frasca, V. Savona, S. Felicetti, R. di Candia, P. Scarlino, Observation of first- and second-order dissipative phase transitions in a two-photon driven Kerr resonator. *Nat. Commun.* **16**, 1954 (2025).

46. K. Seibold, O. Ameye, O. Zilberberg, Floquet expansion by counting pump photons. *Phys. Rev. Lett.* **134**, 060401 (2025).

47. M. Soriente, R. Chitra, O. Zilberberg, Distinguishing phases using the dynamical response of driven-dissipative light-matter systems. *Phys. Rev. A* **101**, 023823 (2020).

48. T. Senthil, Symmetry-protected topological phases of quantum matter. *Annu. Rev. Condens. Matter Phys.* **6**, 299–324 (2015).

49. D. K. J. Boneß, W. Belzig, M. I. Dykman, Resonant-force-induced symmetry breaking in a quantum parametric oscillator. *Phys. Rev. Res.* **6**, 033240 (2024).

50. P. Breiding, M. Michałek, L. Monin, S. Telen, The algebraic degree of coupled oscillators. arXiv:2208.08179 [math.AG] (2022); https://doi.org/10.48550/arXiv.2208.08179.

51. V. Borovik, P. Breiding, J. del Pino, M. Michałek, O. Zilberberg, Khovanskii bases for semimixed systems of polynomial equations - Approximating stationary nonlinear Newtonian dynamics. *J. Math. Pures. Appl.* **182**, 195–222 (2024).

52. V. P. Flynn, E. Cobanera, L. Viola, Deconstructing effective non-Hermitian dynamics in quadratic bosonic Hamiltonians. *New J. Phys.* **22**, 083004 (2020).

53. J. del Pino, J. J. Slim, E. Verhagen, Non-Hermitian chiral phononics through optomechanically induced squeezing. *Nature* **606**, 82–87 (2022).

54. T. Dai, Y. Ao, J. Mao, Y. Yang, Y. Zheng, C. Zhai, Y. Li, J. Yuan, B. Tang, Z. Li, J. Luo, W. Wang, X. Hu, Q. Gong, J. Wang, Non-Hermitian topological phase transitions controlled by nonlinearity. *Nat. Phys.* **20**, 101–108 (2024).

55. N. Pernet, P. St-Jean, D. Solnyshkov, G. Malpuech, N. C. Zambon, Q. Fontaine, B. Real, O. Jamadi, A. Lemaitre, M. Morassi, L. Le Gratiet, T. Baptiste, A. Harouti, I. Sagnes, A. Amo, S. Ravets, J. Bloch, Gap solitons in a one-dimensional driven-dissipative topological lattice. *Nat. Phys.* **18**, 678–684 (2022).

56. L. J. Maczewsky, M. Heinrich, M. Kremer, S. K. Ivanov, M. Ehrhardt, F. Martinez, Y. V. Kartashov, V. V. Konotop, L. Torner, D. Bauer, A. Szameit, Nonlinearity-induced photonic topological insulator. *Science* **370**, 701–704 (2020).

57. T. Inagaki, Y. Haribara, K. Igarashi, T. Sonobe, S. Tamate, T. Honjo, A. Marandi, P. L. McMahon, T. Umeki, K. Enbutsu, O. Tadanaga, H. Takenouchi, K. Aihara, K.-I. Kawarabayashi, K. Inoue, S. Utsunomiya, H. Takesue, A coherent Ising machine for 2000-node optimization problems. *Science* **354**, 603–606 (2016).

58. D. Marković, A. Mizrahi, D. Querlioz, J. Grollier, Physics for neuromorphic computing. *Nat. Rev. Phys.* **2**, 499–510 (2020).

59. M. D. McDonnell, D. Abbott, What is stochastic resonance? Definitions, misconceptions, debates, and its relevance to biology. *PLOS Comput. Biol.* **5**, e1000348 (2009).

60. F. Ragone, J. Wouters, F. Bouchet, Computation of extreme heat waves in climate models using a large deviation algorithm. *Proc. Natl. Acad. Sci. U.S.A.* **115**, 24–29 (2018).

61. Z. Qiao, Y. Lei, N. Li, Applications of stochastic resonance to machinery fault detection: A review and tutorial. *Mech. Syst. Signal. Process.* **122**, 502–536 (2019).

62. L. Gammaitoni, A. R. Bulsara, Noise activated nonlinear dynamic sensors. *Phys. Rev. Lett.* **88**, 230601 (2002).

63. S. R. Rodriguez, Enhancing the speed and sensitivity of a nonlinear optical sensor with noise. *Phys. Rev. App.* **13**, 024032 (2020).

64. M. C. Marchetti, J. F. Joanny, S. Ramaswamy, T. B. Liverpool, J. Prost, M. Rao, R. A. Simha, Hydrodynamics of soft active matter. *Rev. Mod. Phys.* **85**, 1143–1189 (2013).

65. A. Grimm, N. E. Frattini, S. Puri, S. O. Mundhada, S. Touzard, M. Mirrahimi, S. M. Girvin, S. Shankar, M. H. Devoret, Stabilization and operation of a Kerr-cat qubit. *Nature* **584**, 205–209 (2020).

66. A. V. Bolsinov, A. T. Fomenko, *Integrable Hamiltonian Systems: Geometry, Topology, Classification* (CRC Press, 2004).

67. Y. Zhou, J. Lazovskis, M. J. Catanzaro, M. Zabka, B. Wang, "Combinatorial exploration of Morse-Smale functions on the sphere via interactive visualization," in *2023 Topological Data Analysis and Visualization (TopoInVis)* (IEEE Computer Society, 2023), pp. 51–60.

68. M. M. Peixoto, Structural stability on two-dimensional manifolds. *Topology* **1**, 101–120 (1962).

69. M. M. Peixoto, On the classification of flows on 2-manifolds, *Proceedings Symposium Dynamical Systems* (Academic New York, 1973), pp. 389–419.

70. E. Mathieu, Mémoire sur le mouvement vibratoire d'une membrane de forme elliptique. *Journalde Mathématiques Pures et Appliquées* **13**, 137–203 (1868).

71. N. W. Mac Lachlan, *Theory and Application of Mathieu Functions* (Dover, 1964).

72. J. Kosata, A. Leuch, T. Kästli, O. Zilberberg, Fixing the rotating-wave approximation for stronglydetuned quantum oscillators. *Phys. Rev. Res.* **4**, 033177 (2022).

73. M. Aspelmeyer, T. J. Kippenberg, F. Marquardt, Cavity optomechanics. *Rev. Mod. Phys.* **86**, 1391–1452 (2014).

74. L. Papariello, O. Zilberberg, A. Eichler, R. Chitra, Ultrasensitive hysteretic force sensing withparametric nonlinear oscillators. *Phys. Rev. E* **94**, 022201 (2016).

75. P. Breiding, S. Timme, Homotopycontinuation.jl: A package for homotopy continuation in Julia, in *Lect. Notes Comput. Sci.* (including Subser. Lect. Notes Artif. Intell. Lect. Notes Bioinformatics), (Springer, 2018), vol. 10931 LNCS (2018), pp. 458–465, 10.1007/978-3-319-96418-8_54.

76. J. Košata, J. del Pino, T. L. Heugel, O. Zilberberg, HarmonicBalance.jl: A Julia suite fornonlinear dynamics using harmonic balance. SciPost Phys. Codebases, 6 (2022).

77. C. Rackauckas, Q. Nie, DifferentialEquations.jl–A performant and feature-rich ecosystem for solving differential equations in Julia. *J. Open Res. Softw.* **5**, 15 (2017).
